# AMP-activated protein kinase (AMPK) as a potential therapeutic target independent of PI3K/Akt signaling in prostate cancer

**DOI:** 10.18632/oncoscience.49

**Published:** 2014-06-04

**Authors:** Yashmin Choudhury, Zichu Yang, Imran Ahmad, Colin Nixon, Ian P. Salt, Hing Y. Leung

**Affiliations:** ^1^ Institute of Cancer Sciences, University of Glasgow, Glasgow, UK; ^2^ Beatson Institute for Cancer Research, Glasgow, UK; ^3^ Current address - Department of Biotechnology, Assam University, Silchar, India; ^4^ Institute of Cardiovascular and Medical Sciences, University of Glasgow, Glasgow, UK

**Keywords:** Prostate cancer, AMP-activated kinase (AMPK), PI3K, 5-aminoimidazole-4-carboxamide riboside (AICAR), A-769662

## Abstract

Depletion of cellular energy activates the AMP-activated kinase (AMPK) to favor energy-producing catabolic processes during tumorigenesis. Using a panel of *in vitro* cell lines and resected tumors, we investigated the therapeutic value of manipulating AMPK in prostate cancer (PC). Phospho-AMPK expression was significantly elevated in human PC cells and clinical PC samples. In clinical PC, we observed a trend for increasing phospho-AMPK with increasing Gleason sum score; Phospho-AMPK expression was associated with phospho-ACC (p=0.0023). Using the paired PC3 and PC3M cells to model progressive androgen-independent PC, treatment with either 5-aminoimidazole-4-carboxamide riboside (AICAR) or A-769662 suppressed proliferation, migration and invasion in both cell lines, and down-regulated mTOR and P70S6Ki levels regardless of the Akt status. Involvement of AMPK was confirmed by Compound C (AMPK inhibitor) and siRNA-mediated AMPK silencing. Despite similar functional responses in PC3 and PC3M cells, AMPK activation resulted in sustained phospho-Akt activation in PC3M cells, but not in PC3 cells. This prompted the addition of the PI3K inhibitor LY-294002 to AICAR treatment of PC3M cells in a proliferation assay. Interestingly, we found no synergistic effects upon combined treatment. Collectively, these findings support AMPK as a potential therapeutic target independent of PI3K/Akt signalling.

## INTRODUCTION

The AMP-activated protein kinase (AMPK) is a cellular energy sensor comprising of a catalytic α subunit and regulatory β and γ subunits [1]. It is activated in response to metabolic stresses that increase the AMP/ATP ratio either by increasing the consumption of ATP or interfering with the catabolic production of ATP [1].

AMPK is activated when phosphorylated at Thr172 on the α subunit [1]. Two AMPK kinases have been identified that phosphorylate AMPKα Thr172, namely liver kinase B1 (LKB1) and Ca^2+^/calmodulin-dependent protein kinase kinase β (CaMKKβ) [2, 3]. Increased AMP:ATP allosterically activates AMPK (binding to the AMPKγ subunit) and inhibits dephosphorylation of Thr172 in the presence of constitutive LKB1 activity. The precise phosphatase that inactivates AMPK *in vivo* remains uncertain [4]. In cells expressing CaMKKβ, increased intracellular Ca^2+^ concentrations activate AMPK independent of changes in AMP:ATP [3]. Agents that activate AMPK include metformin and phenformin, which increase the AMP:ATP ratio, the nucleoside AICAR, which is metabolised to an AMP mimetic, and A769662, which is a direct activator of AMPK [5].Once activated, AMPK phosphorylates multiple downstream catabolic targets to promote fatty acid oxidation and glucose uptake, while anabolic processes such as fatty acid synthesis and protein synthesis tend to be suppressed [4].

The malignant phenotype in cancer is characterised by increased synthesis of lipid, DNA and protein synthesis as well as enhanced proliferation and migration; AMPK has been shown to be a key regulator of these events [6]. In addition, AMPK can regulate apico-basal cellular polarity of the epithelium, and also directly interact with components of the cell cycle machinery such as centrosomes and spindle poles to control the cell cycle [7, 8]. Cell migration can also be directly controlled by AMPK-mediated phosphorylation of the microtubule plus end protein CLIP-170 [9].

The phosphatidylinositol 3′-kinase (PI3K) signaling network plays critical roles in the regulation of cell growth, proliferation, differentiation, motility, survival and intracellular trafficking. The PI3K/Akt pathway exhibits extensive cross-regulatory interactions with the intermediate metabolism network, including AMPK, to fuel resistance to stress and uncontrolled growth [10]. Akt has been reported to dramatically reduce the AMP/ATP ratio and suppress AMPK activity in cells overexpressing a constitutive Akt mutant [11]. In addition, Akt may directly mediate inhibitory phosphorylation of AMPKα1/α2 at Ser^485/491^ [12]. AMPK may in turn modulate the PI3K pathway in a complex manner, stimulating PI3K/Akt activity and inhibiting mTOR/S6K [13]. mTOR is a component of mTORC1 (mammalian Target of rapamycin Complex 1), which is a key regulator of cell growth, controlling protein synthesis, ribosome biogenesis and autophagy through downstream effectors such as 4EBP1 and S6K1 [14]. In addition to mTOR-mediated regulation of the cell cycle, the mTOR pathway responds to changes in the energy status of the cell, through inhibition of mTORC1 by AMPK [15]. Hence, AMPK/mTOR crosstalk may have potential implications in cancer therapies.

Patients receiving metformin for its hypoglycaemic effects are thought to have a reduced risk of developing cancer [6]. Metformin has *in vitro* anti-proliferative effects on several tumor types including breast, prostate, ovarian, colon and pancreas [6]. However, the role of AMPK in prostate cancer (PC) remains to be fully characterised, with significant discrepancies in the literature. In DU145 cells, a human PC line with activated Akt and upregulated glycolysis, AICAR-mediated AMPK activation suppressed proliferation without evidence of increased apoptosis [16]. Supporting a tumor-suppressing role, a dominant negative AMPK mutant or silencing AMPK expression enhanced proliferation, migration and anchorage independent growth in C4-2 cells, a derived sub-line from human LNCaP PC cells [3]. In contrast, consistent with a tumor-promoting role for AMPK, ~40% of clinical PC showed upregulated levels of phosphorylated acetyl CoA-carboxylase (ACC), which signifies increased AMPK function, while inhibition or depletion of AMPK impaired proliferation and promoted cell death [17].

Given the complex nature of AMPK and PI3K signaling and potential crosstalk between these key pathways, we applied *in vitro* cell models and resected prostate tumors to investigate the role of AMPK and PI3K signaling in prostate cancer.

## RESULTS

### *In vitro* and *in vivo* analysis of the AMPK and PI3K/mTOR pathways in prostate cancer (PC)

**Figure 1:**
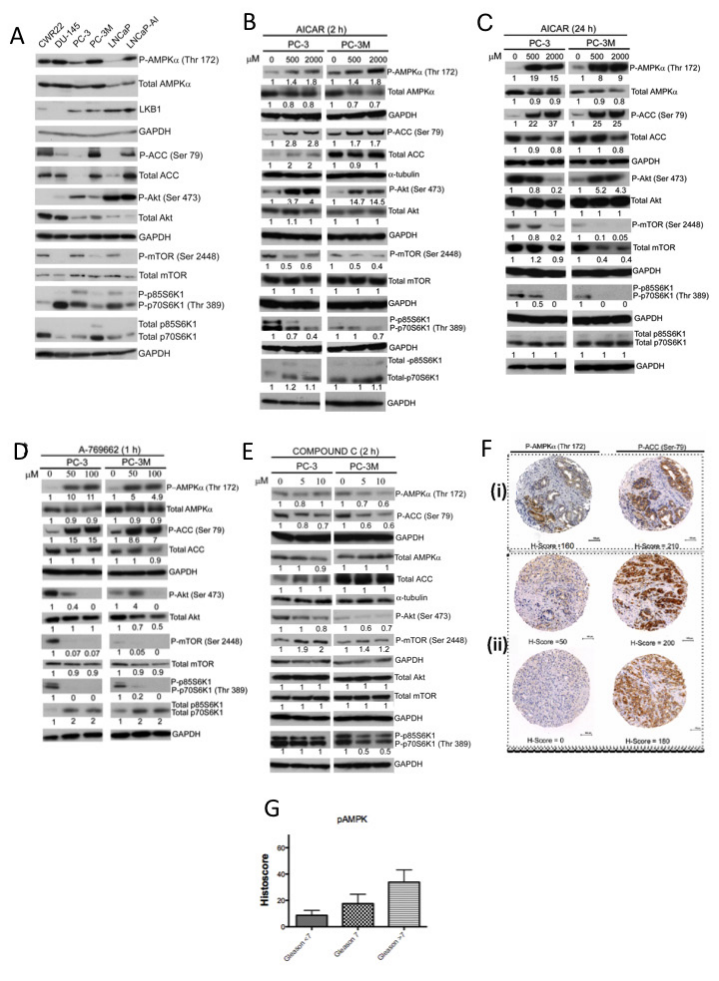
Analysis of the AMPK and PI3K pathways in prostate cancer *in vitro* and *in vivo* (A) Lysates from the indicated prostate cancer cell lines were immunoblotted for phosphorylated and total AMPKα, ACC, Akt, mTOR and p70S6K1 proteins. PC3M and LNCaP-AI were derived from PC3 and LNCaP parental cells, respectively. (B, C, D, E) Whole cell lysates were prepared from PC3 and PC3M cells maintained in serum free medium and treated with (B) AICAR (500 and 2000 μM) for 2 h, (C) AICAR (500 and 2000 μM) for 24 h, (D) A-769662 (50 and 100 μM) for 1 h, and (E) Compound C (5 μM and 10 μM) for 2 h. (F) Representative images showing phospho-AMPKα and phospho-ACC in clinical prostate tumors with (i) high Histoscore (H-Score) for both p-AMPKα and P-ACC and (ii) low H-Score for phospho-AMPKα but high H-Score for phospho-ACC. (Scale bar=100 μm). (G) Trend for increasing phospho-AMPKα immunoreactivity with tumors with high Gleason sum score (<7, 7 and >7), with statistically significant difference between Gleason 8 or higher to Gleason <7 (p=0.0251, Mann-Whitney test). [(B, C, D, E)]. Values under Western blots represent level of each protein normalized to GAPDH or α-tubulin. Western blots (panels A-E) are representative of three independent experiments.

The status of AMPK and PI3K/mTOR pathways was highly varied in a panel of human PC cell lines (Fig. 1A). The highly aggressive PC3M and hormone-independent LNCaP-AI cells exhibited higher levels of AMPK (both total and phosphorylated) and its substrate ACC relative to the parental PC3 and LNCaP cells, respectively. The variation in phospho-AMPK was independent of the level of the upstream kinase LKB1, which was expressed at similar levels in all cell lines except in the LKB1-deficient DU145 cells. While the level of total Akt was higher in PC3M cells than in PC3, there was more phospho-Akt in PC3 cells than PC3M cells, suggesting preferential activation of Akt in PC3 cells compared to PC3M cells. This variation in activation of Akt was, however, not observed between LNCaP and LNCaP-AI cells. Correlating to the phospho-Akt status, the levels of phosphorylated mTOR and its downstream effector p70S6K1 (along with the isoform p85S6K1) were lower in PC3M cells than in PC3 cells. In order to investigate the functional significance related to differences in AMPK and PI3K/mTOR pathways between PC3 and PC3M cells, subsequent *in vitro*experiments in this study focused on PC3 and PC3M cells.

Incubation of PC3 or PC3M cells with either AICAR or A-769662 upregulated the levels of AMPK and ACC phosphorylation (Fig. 1B, C, D). AMPK activation was associated with differential patterns of Akt phosphorylation between PC3 and PC3M cells. While incubation with AICAR for 2 h upregulated phospho-Akt in both PC3 and PC3M cells (Fig. 1B), longer treatment with AICAR for 24 h revealed distinct responses, with similar observation made following A-769662 treatment for 1 h: Akt phosphorylation was stimulated in PC3M cells but attenuated in PC3 cells (Fig. 1 C, D). Furthermore, in PC3M cells, A-769662 treatment (50 μM) increased phospho-Akt, while the higher dose of A-769662 at 100 μM reduced phosphorylation of Akt (Fig. 1D). AICAR and A-769662 downregulated phospho-mTOR^Ser 2448^ in both PC3 and PC3M cells, and, interestingly, total mTOR was downregulated in PC3M cells following AICAR treatment (24 h). Concomitant suppression of p70S6K1 and p85S6K1 phosphorylation was observed following AMPK activation (Fig. 1B, C, D).

To validate the specific nature of our AMPK activators, Compound C (CC), a cell-permeable pyrrazolopyrimidine compound, was used as a reversible ATP-competitive inhibitor of AMPK [18]. As expected, Compound C suppressed the steady state of both AMPK and ACC phosphorylation in normal culture conditions (Fig.1E), along with decreased phospho-Akt and increased phospho-mTOR levels.

Studying AMPKα and ACC phosphorylation status *in vivo*, a tissue microarray (TMA) of resected clinical PC was studied by immunohistochemistry (Fig.1F). Overall, when compared to benign prostate hyperplasia (BPH), PC showed upregulated expression for phospho-AMPKα and phospho-ACC, with mean histo-score of 19.39 (range 0-160, n=123) and 33.61 (range 0-270, n=102) respectively, contrasting with mean histoscore of zero for both phospho-AMPKα and -ACC in BPH samples. Phospho-AMPK immunoreactivity was significantly higher in poorly differentiated tumors (Gleason score 8 or above) than low grade tumors (Gleason score <7), p=0.0251, Mann-Whitney test (Fig. 1G). There was also a significant positive correlation between phospho-AMPKα and phospho-ACC levels (Spearman correlation coefficient = 0.4588; P = 0.0003). Interestingly, in some tumors, we observed significant phospho-ACC immunoreactivity despite low phospho-AMPKα levels (Fig. 1Fii).

### Analysis of AMPK-mediated functions in PC *in vitro*

**Figure 2:**
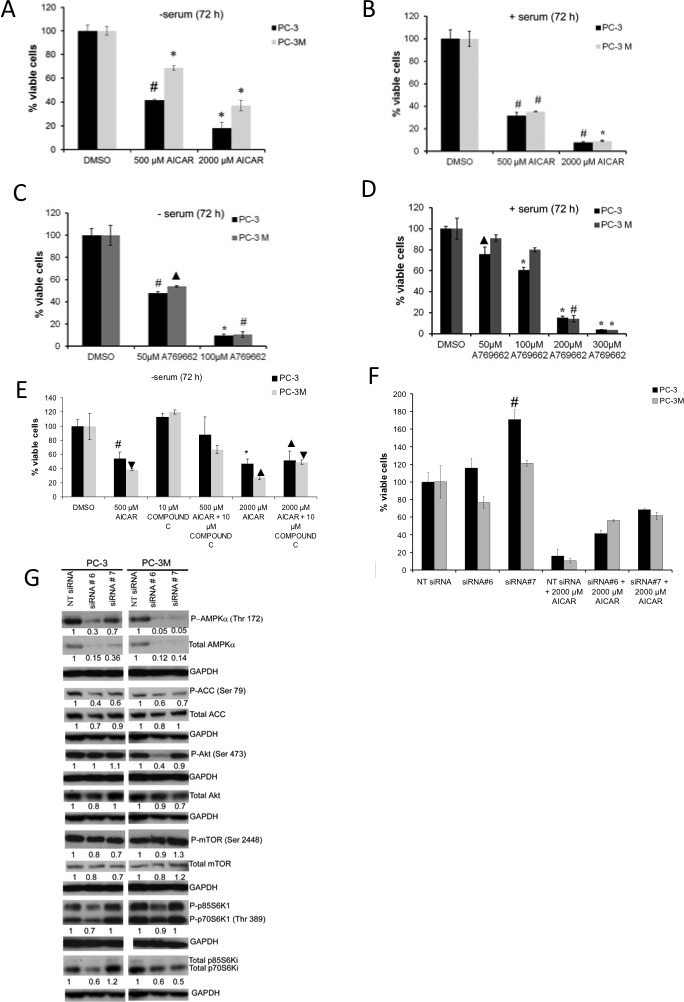
Treatment with AICAR or A-769662 suppressed proliferation in PC3 and PC3M cells Cell proliferation assay of PC3 and PC3M cells following treatment with (A, B) AICAR (500 and 2000 μM) or (C, D) A-769662 (50-300 μM); panels A, C for serum free medium and panels B, D for serum-supplemented medium. (E) Proliferation assay of PC3 and PC3M cells following treatment with AICAR (2000 μM) and Compound C (10 μM) alone or in combination, in serum free medium. Data presented in panels A-E are the values for mean ± SD of cell viability respective to (DMSO) control from three independent experiments, each with three technical replicates. For A through E * indicates significant difference at P ≤ 0.001, # at P ≤ 0.005, ▲ at P ≤ 0.01 and ▼ at P ≤ 0.05 from respective control. (F) PC3 and PC3M cells were incubated with siRNA targeting AMPKα1 alone or with AICAR (2000 μM) prior to cell proliferation assays. Data are presented as mean ± SD of cell viability respective to non-targeting, NT siRNA control from two independent experiments, each with three technical replicates. (G) Whole cell lysates were prepared from PC3 and PC3M cells following incubation with siRNA targeting AMPKα; Values under Western blots represent level of each protein normalized to GAPDH. Western blots are representative of two independent experiments.

AMPK activation by AICAR (500 μM and 2000 μM) or A-769662 (50 μM and 100 μM) significantly suppressed proliferation in both PC3 and PC3M cells in a dose-dependent manner, regardless of the extent of serum supplement in the culture medium (Fig. 2A-D). Interestingly, when cultured in full medium supplement, proliferation of PC3M cells was significantly diminished by A-769662 only at higher concentrations (200 and 300 μM). The involvement of AMPK in the observed anti-mitogenic effects was supported by: (1) Compound C (CC) treatment, at least partially, rescued both PC3 and PC3M cells from AICAR-induced suppression of proliferation (Fig. 2E); (2) Importantly, siRNA-targeting AMPKα1 abolished AICAR induced anti-proliferative effects (Fig. 2F). In PC3 cells, knockdown of AMPK expression has an overall mitogenic effect despite the presence of AICAR; and (3) Treatment with either CC (Fig. 2E,S1) or siRNA-mediated silencing of AMPKα1 expression did not suppress proliferation (Fig. 2F). Following siRNA-mediated AMPK knockdown, both total and phosphorylated states of AMPK and ACC were suppressed. Contrast to treatment with AMPK activators, AMPK knockdown did not upregulate it did not increase the level of phospho-Akt, which was either unchanged or reduced following AMPK gene silencing.(Fig. 2G).

**Figure 3:**
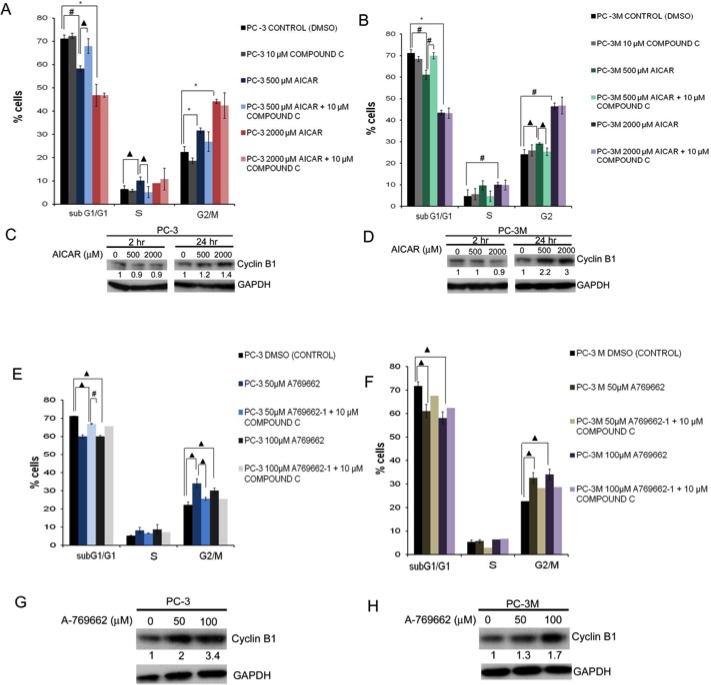
Treatment with AICAR or A-769662 induced G2/M arrest in PC3 and PC3M cells and cyclin B1 accumulation (A, B, E, F) Cell-cycle profile analysis in (A, E) PC3 and (B, F) PC3M cells treated with (A, B) AICAR (500 and 2000 μM) or (E, F) A-769662 (50 and 100 μM) in the presence or absence of Compound C (10 μM). Data of cell-cycle profile analysis were presented as mean ± SD. * indicates significant difference at P ≤ 0.001, # at P ≤ 0.005 and ▲ at P ≤ 0.01. (C, D, G, H) Western blot of whole cell lysates from (C, G) PC3 and (D, H) PC3M cells treated with (C, D) AICAR (500 and 2000 μM) for 2 h and 24 h or (G, H) A-769662 (50 and 100 μM) for 1 h, probed for cyclin B1 protein. Values under Western blots represent level of each protein normalized to GAPDH.

Contrast to a dominant G1 cell fraction in the control PC3 and PC3M cells, AICAR or A-769662 treatment significantly shifted the cell cycle profile towards the G2/M phase (Fig. 3A,B for AICAR; 3E,F for A-769662, respectively). Anti-proliferative effects of AMPK activators (AICAR or A-769662) were not associated with apoptosis, signified by the absence of caspase-3 or PARP cleavage products (Fig. S2). The addition of CC reversed the effects of both AICAR and A-769662 (except for AICAR at the high dose of 2000 μM which probably reflects off-target effects). We also observed cyclin B1 accumulation following AMPK activation, which is consistent with G2/M arrest (Fig. 3C, D; 3G, H). CC treatment alone appeared to have the reverse effect and diminished cyclin B1 expression (Fig. S3A), while siRNA-mediated knockdown of AMPKα1 in isolation resulted in either reduced or mostly unchanged cyclin B1 levels (Fig. S3B).

**Figure 4:**
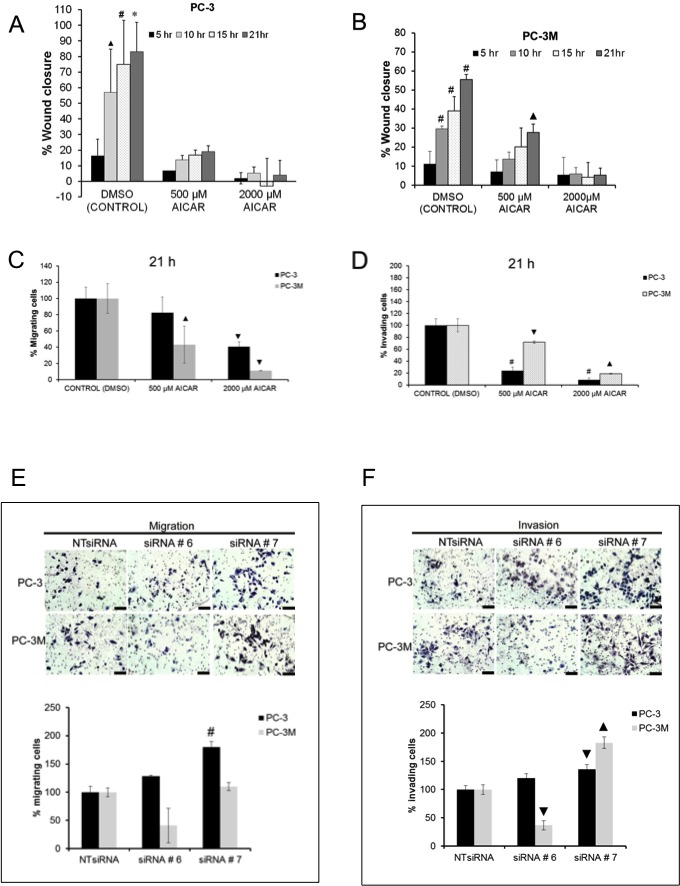
Treatment with AICAR suppressed migration and invasion in PC3 and PC3M cells (A, B) Wound healing assay by time-lapse microscopy showing migration of (A) PC3 and (B) PC3M cells in serum free medium in the presence or absence of AICAR (500 and 2000 μM). Data are presented as mean ± SD of three independent experiments. (C, D) Transwell assay using PC3 and PC3M cells for (C) migration or (D) invasion in the presence of AICAR (500 and 2000 μM) relative to (DMSO) control. (E) Migration of PC3 and PC3M cells through insert membranes in a transwell migration assay after siRNA-mediated knockdown of AMPKα1, relative to those treated with non-targeting siRNA (control), over a period of 21 h. (F) Invasion of PC3 and PC3M cells through Matrigel coated membranes in a transwell invasion assay after si-RNA mediated knockdown of AMPKα1 over a period of 21 h (non-targeting siRNA = control). Scale bar = 100 μM. Three independent experiments were performed for migration and invasion of PC3 and PC3M cells. Data are presented as mean ± SEM of three independent experiments. * indicates significant difference at P ≤ 0.001, # at P ≤ 0.005, ▲ at P ≤ 0.01 and ▼at P ≤ 0.05 from respective control.

As PC3M cells represent an aggressive isogenic cell line derived from PC3 cells, we investigated the effect of AMPK activation on cellular motility and invasion. In a scratch wound assay, AMPK activation by AICAR significantly suppressed migration of both PC3 and PC3M cells in a dose-dependent manner (Fig. 4A, B; S4A, B). Similar results were obtained in a transwell migration assay (Fig. 4C; S4C). Studying *in vitro* invasion through matrigel-coated membranes, AICAR treatment inhibited PC3 and PC3M cellular invasion in a dose-dependent manner; PC3M cells appeared to be less responsive to AICAR at 500 μM than PC3 cells (Fig. 4D; S4D). For PC3 cells, AMPKα1-targeting siRNA enhanced both migration and invasion (Fig. 4 E, F). Interestingly, while PC3M cells were less responsive to AICAR treatment, impact of AMPK gene silencing was also more varied. Taken together, despite differences in the duration of phospho-Akt activation in PC3 and PC3M cells following AMPK activation, both cell lines responded similarly to AICAR treatment with reduced proliferation, migration and invasion.

### AMPK-mediated anti-proliferative effect in PC3M cells is independent of PI3K/Akt signaling

**Figure 5:**
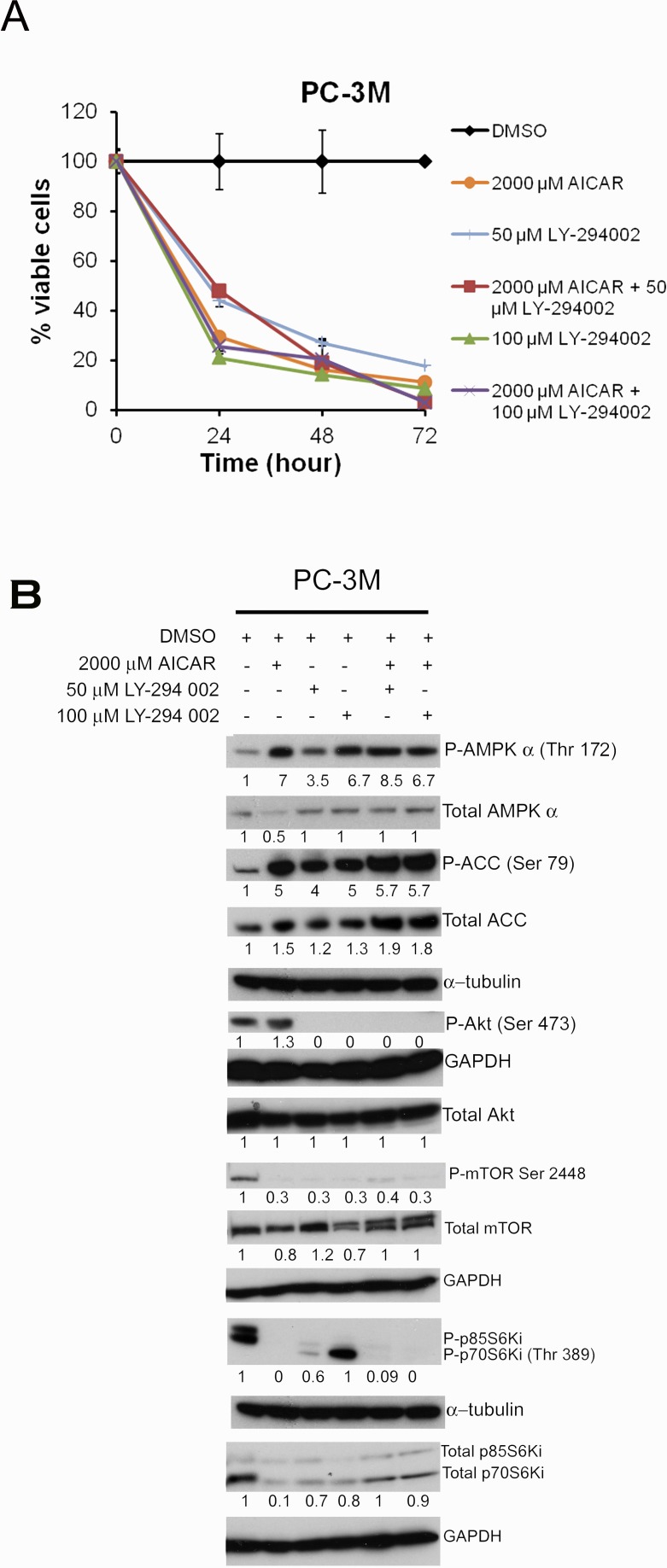
Treatment with AICAR or LY-294002 alone or in combination in PC3M cells (A) Cell proliferation assay for PC3M cells treated with AICAR (2000 μM) or LY-294002 (50 and 100 μM) alone or in combination, for 24 h, 48 h and 72 h respectively. Data are presented as mean ± SD relative to viability at the start of the assay. (B) Western blot analysis of PC3M cells treated with the indicated concentrations of AICAR or LY-294002 alone or in combination for 24 h. Values represent levels of each protein normalized to GAPDH. The blots are representative of Western blots from three independent experiments.

To test the significance of phospho-Akt induction by AICAR (Fig. 1B, C), AICAR treatment was combined with the PI3K inhibitor LY-294002 in a proliferation assay using PC3M cells (Fig. 5). As expected, either AICAR or LY-294002 alone suppressed proliferation in PC3M cells (Fig. 5A). However, combined AICAR and LY-294002 treatment did not produce additional anti-proliferative effects on PC3M cells. LY-294002 treatment alone increased both AMPK and ACC phosphorylation in a dose-dependent manner, and, as a result of PI3K suppression, abolished Akt and mTOR phosphorylation (Fig.5B). In the presence of LY-294002 treatment, the effects of AICAR on Akt/mTOR phosphorylation were not observed. Hence, AICAR-induced phospho-Akt activation (Fig. 1B & C) did not appear to have a pro-survival effect and may merely reflect a compensatory response or an off-target effect (Fig. 5 A).

## DISCUSSION

To date, the role of AMPK in prostate cancer (PC) remains unclear [6]. In this study, while phospho-AMPK and phospho-ACC immunoreactivities were significantly upregulated in resected PC, particularly in high-grade tumors, *in vitro* activation of AMPK resulted in tumor-suppressing effects (reduced proliferation, migration and invasion) regardless of the background AMPK status and culture conditions (with and without serum supplement). In clinical PC, the presence of high phospho-ACC without correspondingly elevated phospho-AMPK level may suggest AMPK-independent phosphorylation of ACC, reduced ACC phosphatase activity or significant allosteric activation of AMPK. Consistent with enhanced phospho-AMPK status in PC, AMPK mRNA expression was also found to be altered in 42 of 131 (or 32%) primary tumours with mRNA, majority of which showed upregulated AMPK expression (Figure S5).

Using isogenic pairs of human PC cells derived from PC3 and LNCaP cell lines, we found increasing levels of phospho-AMPK and phospho-ACC in the aggressive PC3M and LNCaP-AI sub-cells, supporting the association between high phospho-AMPK levels in aggressive clinical disease. We also characterised the impact of chemical activation of AMPK on the PI3K/Akt/mTOR signaling pathway. Comparing PC3 and PC3M cells, phosphorylation of Akt by AMPK agonists (AICAR or A-769662) was more sustained in PC3M cells. Despite this difference between PC3 and PC3M cells, functional analyses for cellular proliferation, cell cycle profile, migration and invasion showed broadly similar responses in both cell lines upon AMPK activation or suppression. Overall, our data suggest AMPK-mediated effects to be independent of phospho-Akt. Besides the PI3K/Akt pathway, another actionable interaction has been suggested: AMPK inhibition in DU145 cells enhanced Fas-induced apoptosis via ubiquitination-mediated proteasomal degradation of c-FLIP, an apoptosis inhibitory protein [20].

Accepting the limitation of chemical modulators of AMPK with evidence of non-specific and off-target effects for both AICAR and Compound C [21, 22, 23], data from our siRNA-mediated silencing of AMPK support the involvement of AMPK in our phenotypic observations. Activation of AMPK by either AICAR or A-769662 downregulated phospho- mTOR and phospho-p70S6K, irrespective of Akt phosphorylation in a non-canonical manner [15]. Park et al have previously suggested that the decreased proliferation in response to AICAR resulted from non-specific effect on nucleotide metabolism [24]. In keeping with AMPK's role as a metabolic sensor, anti-mitotic effects following AICAR activation was associated with suppressed *de novo* fatty acid synthesis, evidenced by reduced ACC and fatty acid synthase expression along with lower levels of the ACC product malonyl CoA [25].

AMPK activation has been reported to upregulate the p53–p21 axis to bring about cell cycle arrest [26]. Interestingly, we observed that AMPK activation by AICAR or A-769662 induced G2/M arrest in the p53-null PC3 and PC3M cells, with the AMPK inhibitor Compound C at least partially reversed this effect. AICAR or A-769662 mediated AMPK activation was associated with upregulated cyclin B1 expression in a time- and dose-dependent manner. The failure of cyclin B1 to fall may prevent progression through the G2/M checkpoint [27]. We observed no evidence of enhanced apoptosis in PC3 and PC3M cells upon AMPK activation, consistent with that previously reported [16].

PC3 cells (and the derived aggressive PC3M cell line) are negative for the tumor suppressor PTEN, a critical negative regulator of the PI3K pathway, leading to constitutive Akt activity [28]. In our study using PC3 and PC3M cells, different AMPK activators (AICAR or A-769662) induced phospho-Akt for different durations in the cell lines (Figure 1). Simultaneous AICAR and LY-294002 (PI3K inhibitor) treatment in PC3M cells did not result in additional reduction of proliferation. Therefore, induction of phospho-Akt by AMPK is unlikely to have a major role as a pro-survival signal. Since A-769662 is reported to be a more efficient activator of AMPK [29], differences in the profile of phospho-Akt following treatment with AMPK-activators (AICAR or A-769662) may reflect the pharmacodynamics of these agents, with a relatively slower response to AICAR when compared to A-769662. Prior to formal exploitation of AMPK targeting therapy in PC in the future, it is therefore necessary to probe for relevant interactions between networks driving invasive PC.

## Materials and methods

### Cell culture and treatment

Human prostate cancer cell lines CWR22, DU-145, PC3, PC3M, LNCaP, and LNCaP-AI were authenticated by LCG standards. Cells were grown in RPMI 1640 medium (Gibco) containing 10 % (v/v) serum supplement and 2 mM L-Glutamine in a humidified incubator (37°C and 5 % CO_2_). All incubations in the presence or absence of various compounds or siRNA were performed in a humidified incubator (37°C and 5 % CO_2_). The AMPK inhibitor Compound C {6-[4-(2-Piperidin-1-yl-ethoxy)-phenyl)]-3-pyridin-4-yl-pyrrazolo[1,5-a]-pyrimidine} was obtained from Calbiochem and stored as a 10 mM solution in DMSO at 2-8°C, until further use. Prior to treatment, sub-confluent cultures of PC3 and PC3M cells were incubated in serum free medium comprising RPMI 1640 and 2 mM L-Glutamine for 2 h, following which AICAR (Aminoimidazole-4-carboxamide-1-β-D-ribofuranoside, Sigma-Aldrich), Compound C (Calbiochem), or the solvent of these compounds, DMSO (control) were added in serum free medium and the cells incubated for 2 h and 24 h respectively. Treatment with LY-294002 (Sigma-Aldrich) alone or in combination with AICAR was performed in a similar manner for 24 h. Treatment with A-769662 (Abcam) was preceded by incubation of PC3 and PC3M cells in serum free medium for 1 h, following which A-769662 or DMSO (control) were added, and the cells were incubated further for 1 h.

### si-RNA mediated knockdown of AMPKα

ON-TARGET plus PRKAA1 siRNA J-005027-06 and J-005027-07 (referred to as siRNA #6 and #7, respectively, thereafter)(Dharmacon) were used for knockdown of AMPKα1. ON-TARGET plus non-targeting siRNA#1 (Fermentas) was used as a negative control. siRNAs were transfected into PC3 and PC3M cells in medium supplemented with serum using Lipofectamine RNAiMax (Invitrogen) according to manufacturer's instructions, following which cells were incubated at 37°C and 5 % CO_2_ for 72 h.

### Western blot analysis

Cells were lysed in lysis buffer [50 mM Tris-HCl pH 7.6, 150 mM sodium chloride, 1% (v/v) triton-X 100, 0.5% (v/v) deoxycholate, 0.1% (v/v) SDS, 1 mM sodium orthovanadate, 5 mM sodium fluoride, 50 mg/L phenyl methyl sulphonyl fluoride, 1x protease inhibitor cocktail mix (Calbiochem) and 1x PhosSTOP (Roche)]. The protein samples were separated by SDS-PAGE and transferred to Immobilon P polyvinylidenedifluoride membrane (Millipore). The membranes were then blocked with 5% non-fat dry milk and incubated in a solution of specific primary antibody at dilution 1:1000. The primary antibodies used are listed below: rabbit anti-AMPKα (#2603), anti-phospho-AMPKα (Thr 172) (#2535), anti-ACC (#3676), anti-phospho-ACC (Ser 79) (#3661), anti-Akt (#9272), anti-phospho-Akt (Ser 473) (#4060), anti-mTOR (#2983), anti-phospho-mTOR (Ser 2448) (#2976), anti-p70S6K (#9202), anti-phospho-p70S6K (Thr 389) (#9234) and anti-caspase 3 (#9662) antibodies from Cell Signaling Technology; anti-cyclin B1 from Abcam; mouse anti-PARP (4338-MC-50) antibody from Genzyme; mouse anti-α-tubulin (sc-8035) antibody from Santa Cruz Biotechnology and Horseradish Peroxidase (HRP)-labeledmouse anti-GAPDH (Sigma-Aldrich). Secondary antibody used was HRP-labelled goat anti-rabbit IgG or horse anti-mouse IgG (Cell Signaling) at a dilution of 1:5000. Chemiluminescent detection was performed using ECL Western Blotting Reagent (Amersham) according to manufacturer's instructions.

### Cell proliferation assay

PC3 and PC3M cells (5×10^3^/well) were plated in a 96-well plate in triplicate, and grown in medium supplemented with serum for 24 h in a humidified incubator (37°C and 5% (v/v) CO_2_), following which the required compounds, DMSO (control) or siRNA were added, and the cells incubated for further 24, 48 or 72 h. At the end of the period of treatment, the numbers of viable cells were determined using Cell Proliferation Reagent WST-1 (Roche), as described by the manufacturer.

### Cell cycle analysis

PC3 and PC3M (1x10^6^) were grown in medium supplemented with serum for 24 h, following which the compound(s) of interest or DMSO (control) were added, and the cells incubated for 72 h. Subsequently, the cells were harvested, fixed in 70 % ethanol and stained with propidium iodide (10μg/ml) after treatment with RNase (400 μg/ml). The cell cycle profiles were acquired using a flow cytometer (FACSCalibur, BD) and analysed using Cell Quest Pro software.

### Scratch wound assay

The scratch wound assay was modified from a previously reported protocol [18]. PC3 and PC3M cells were grown to a confluent monolayer in medium supplemented with serum, following which they were incubated in serum-free medium for 2 h at 37°C and 5% (v/v) CO_2_. Three scratches per well were made in the monolayer using a P250 micropipette tip. The study compounds or DMSO (control) were added to each well in serum free medium. The migration of cells into the wound was then visualized using a Long Term Time Lapse Microscope (Nikon), over a period of 21 h.

### Transwell migration and invasion assays

The assay for migration of PC3 and PC3M cells was performed in 24-well plates across 8 μm Cell Culture Inserts (BD Falcon). Briefly, 5×10^4^/well PC3 or PC3M cells were suspended in serum free medium containing the required compounds or DMSO (control) and allowed to migrate across the membrane of the inserts, using medium supplemented with serum and containing the same concentration of the compounds or DMSO, as a chemo-attractant. Conversely, 5×10^4^ PC3 or PC3M cells transfected with specific siRNA targeting AMPKα1 were suspended in serum free medium, and allowed to migrate across the membrane of the inserts using only medium supplemented with serum as a chemo-attractant. The cells were incubated in a humidified chamber at 37°C and 5% CO_2_, and allowed to migrate over a period of 21 h.

The assay for PC3 and PC3M invasion was performed using Biocoat Matrigel Invasion Chambers (BD), as described for the transwell migration assay.

### Expression analysis in resected prostate cancer specimens

A tissue microarray (TMA) consisting of formalin-fixed and paraffin-embedded prostate cancer (PC, n=209) and benign prostatic hyperplasia (BPH, n=30) samples was generated and used for immunohistochemical analysis. Briefly, sections from the TMA were deparaffined, rehydrated and antigen retrieval performed by incubation in citrate buffer (10mM, pH 6) at 98°C, followed by incubation in blocking solution containing 5% (v/v) goat serum. Tissue sections were then incubated in solutions containing the relevant primary antibodies (rabbit anti-phospho-AMPKα (Thr^172^) and anti-phospho-ACC (Ser^79^) antibodies) overnight at 4°C at dilutions of 1:75 and 1: 500 respectively, followed by incubation in HRP-labelled secondary antibody for 1 h at room temperature (Dako Envision System) was used. Negative controls were included with the primary antibody omitted prior to incubation in the secondary antibody. 3,3′-Diaminobenzidine (Ultravision Detection System, Thermo Scientific) was used as the chromogen, and hematoxylin as counterstain. The cores were blindly scored by two independent observers (YC and IA) to generate a Histoscore (H-Score), defined as intensity of staining, quantified as 0 for undetectable staining, 1 for low staining, 2 for intermediate staining and 3 for high staining multiplied by the area of the core stained with a specific intensity [19].

### Statistical Analysis

All data is represented as mean ± SD, with the exception of data pertaining to the Transwell migration assay and invasion assay, which is represented as mean ± SEM. Statistical significance was assessed by Student's *t* test (2-tailed).

## SUPPLEMENTARY FIGURES


