# Supplementary material for: AMP-activated protein kinase (AMPK) as a potential therapeutic target independent of PI3K/Akt signaling in prostate cancer
Source: Oncoscience. 2014 Jun 4;1(6):446–56. doi: 10.18632/oncoscience.49 (PMC4284621; doi:10.18632/oncoscience.49)
Supplement: Supplementary file 1 [file oncoscience-01-0446-s001.pdf]

# AMP-activated kinase (AMPK) as a potential therapeutic target independent of PI3K/Akt signaling in prostate cancer

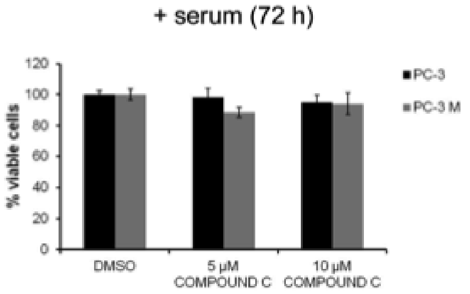


**Figure S1: Treatment with Compound C (5 and 10 µM) to inhibit AMPK did not affect proliferation of PC3 and PC3M cells in serum-free culture condition.** Data are presented as mean ± SD of cell viability respective to control for three independent experiments, with three technical replicates each.

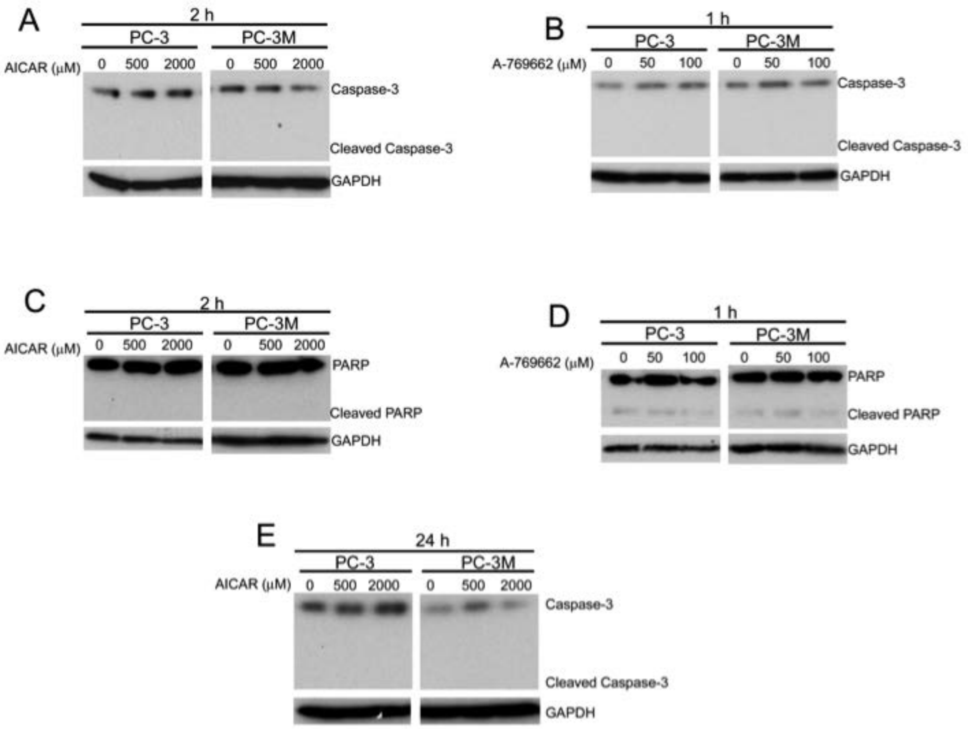


**Figure S2. AICAR or A-769662 treatment does not lead to cleavage of caspase-3 or PARP**. Western blot of PC3 and PC3M cell lysates following treatment with AICAR or A-769662 probed for caspase-3 (A, B, E) and PARP (C, D). The blots are representative of Western blots from three independent experiments.

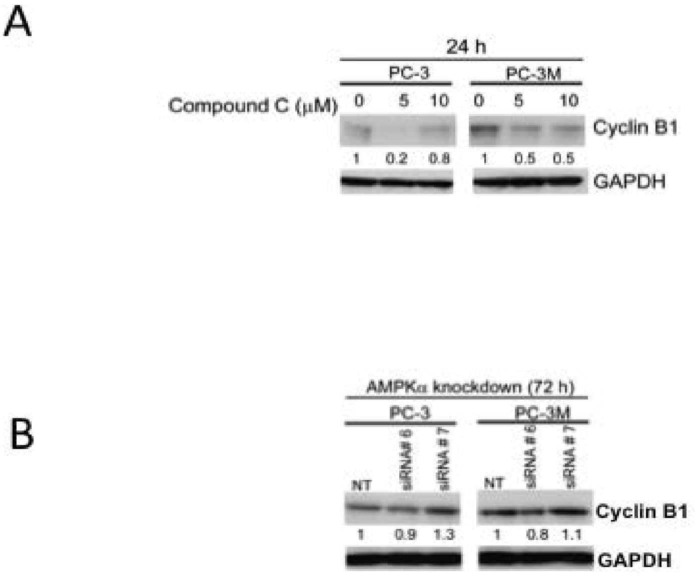


**Figure S3: Inhibition of AMPK did not increase cyclin B1 levels in PC3 and PC3M cells**. Western blots of whole cell lysates from PC3 and PC3M cells were probed for cyclin B1 following (A) treatment with Compound C (5 and 10 µM) for 24 h, or (B) incubation with siRNA targeting AMPKα1 expression. Values represent the level of each protein normalised to GAPDH. The blots are representative of Western blots from three independent experiments.

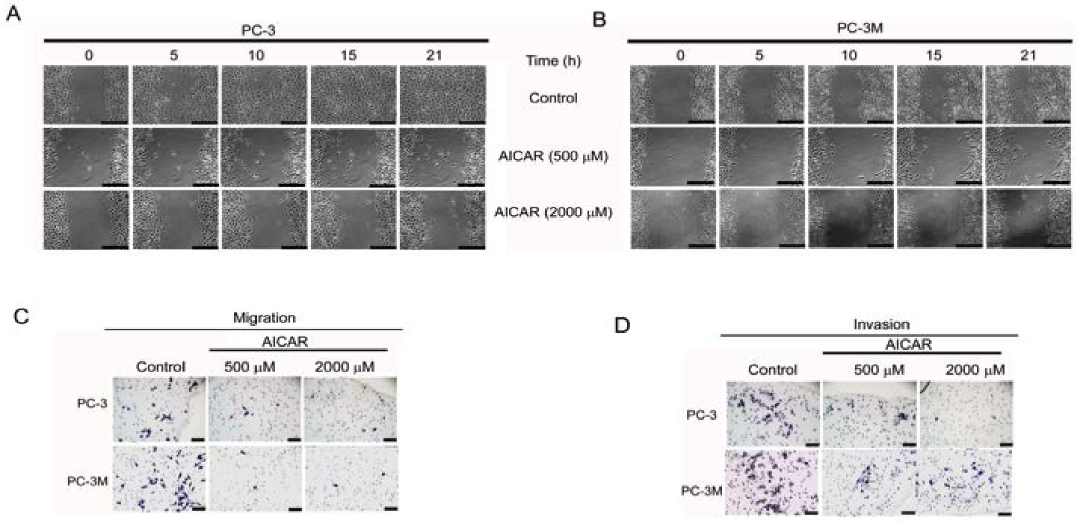


**Figure S4**. **Treatment with AICAR suppressed migration and invasion of PC3 and PC3M cells.** Representative images from wound closure assays showing migration of (A) PC3 and (B) PC3M cells in serum free medium in the presence or absence of AICAR (500 or 2000 µM). (C) Migration of PC3 and PC3M cells through transwell membranes or (D) Invasion of PC3 and PC3M cells through Matrigel-coated membranes in the presence or absence of AICAR over a period of 21 h.

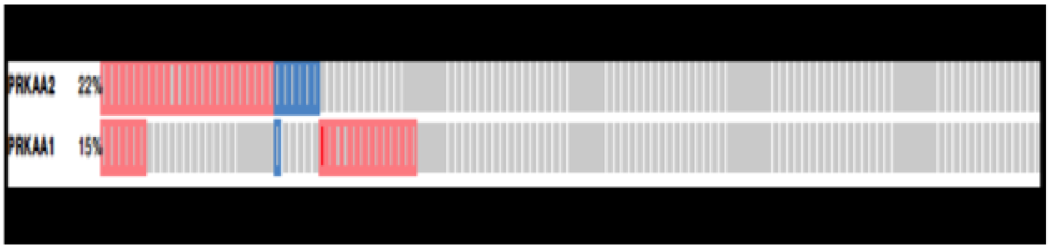


Case Set: Primary Tumors with mRNA: All primary tumor samples with mRNA expression data (131 samples). Altered in 42 (32%) of cases (red and blue bars signify upregulated and dowregulated expression in PC, respectively).

**Figure S5. Analysis of AMPK expression in clinical prostate cancer cohort in cBio portal dataset (http://www.cbioportal.org).**
